# The Role of Celestial Compass Information in *Cataglyphis* Ants during Learning Walks and for Neuroplasticity in the Central Complex and Mushroom Bodies

**DOI:** 10.3389/fnbeh.2017.00226

**Published:** 2017-11-14

**Authors:** Robin Grob, Pauline N. Fleischmann, Kornelia Grübel, Rüdiger Wehner, Wolfgang Rössler

**Affiliations:** ^1^Behavioral Physiology and Sociobiology (Zoology II), Biozentrum, University of Würzburg, Würzburg, Germany; ^2^Brain Research Institute, University of Zürich, Zürich, Switzerland

**Keywords:** look-back behavior, desert ants, vector navigation, sky-compass pathway, memory, central complex, mushroom body, visual orientation

## Abstract

Central place foragers are faced with the challenge to learn the position of their nest entrance in its surroundings, in order to find their way back home every time they go out to search for food. To acquire navigational information at the beginning of their foraging career, *Cataglyphis noda* performs learning walks during the transition from interior worker to forager. These small loops around the nest entrance are repeatedly interrupted by strikingly accurate back turns during which the ants stop and precisely gaze back to the nest entrance—presumably to learn the landmark panorama of the nest surroundings. However, as at this point the complete navigational toolkit is not yet available, the ants are in need of a reference system for the compass component of the path integrator to align their nest entrance-directed gazes. In order to find this directional reference system, we systematically manipulated the skylight information received by ants during learning walks in their natural habitat, as it has been previously suggested that the celestial compass, as part of the path integrator, might provide such a reference system. High-speed video analyses of distinct learning walk elements revealed that even exclusion from the skylight polarization pattern, UV-light spectrum and the position of the sun did not alter the accuracy of the look back to the nest behavior. We therefore conclude that *C. noda* uses a different reference system to initially align their gaze directions. However, a comparison of neuroanatomical changes in the central complex and the mushroom bodies before and after learning walks revealed that exposure to UV light together with a naturally changing polarization pattern was essential to induce neuroplasticity in these high-order sensory integration centers of the ant brain. This suggests a crucial role of celestial information, in particular a changing polarization pattern, in initially calibrating the celestial compass system.

## Introduction

Before starting their foraging career central place foragers, like bees, wasps and ants, have to acquire knowledge about the position of their nest in its surroundings and need to calibrate their navigational toolkit (Collett et al., 2013; Fleischmann et al., 2016). In order to do so, they perform learning flights or walks. Studies of this early learning behavior in bees (Opfinger, 1931; Hempel de Ibarra et al., 2009; Philippides et al., 2013; Degen et al., 2015), wasps (Zeil et al., 1996; Stürzl et al., 2016) and ants (Wehner et al., 2004; Fleischmann et al., 2016, 2017) revealed striking parallels in the general sequence of this behavior (Zeil, 2012). When leaving the nest entrance for the first time honeybees (Lehrer, 1993), bumblebees (Hempel de Ibarra et al., 2009; Collett et al., 2013; Philippides et al., 2013) and wasps (Zeil et al., 1996; Stürzl et al., 2016) turn back immediately towards their nest entrance and look back before flying in multiple arcs parallel to the nest entrance. As walking insects do not walk sideways, ants perform repeated turns during their learning walk loops and make stops to look back towards their nest entrance (Wehner et al., 2004; Fleischmann et al., 2017). During these looks back the animals most probably learn the landmark panorama (honeybees: Opfinger, 1931; Lehrer, 1993; bumblebees: Collett et al., 2013; ants: Fleischmann et al., 2016, 2017). Over time the arcs or loops increase in size, and novices move farther away from the nest entrance, while still looking back towards it (Zeil et al., 1996; Wehner et al., 2004; Philippides et al., 2013; Fleischmann et al., 2016). Likewise, experienced foragers perform a learning behavior that includes looks back to the nest, e.g., when experienced animals had difficulties pinpointing their nest (Zeil, 1993; Zeil et al., 1996) or when the nest surrounding had changed drastically (Müller and Wehner, 2010; Narendra and Ramirez-Esquivel, 2017).

However, to determine the direction of the nest entrance from various positions in space, the animals need some kind of reference system. It has been previously proposed, that this system could be part of the path integrator (Graham et al., 2010; Müller and Wehner, 2010), which integrates information about the walked directions (compass) and the distance covered (odometer) into a vector pointing towards the starting point. In *Cataglyphis* ants the path integrator is the main navigational tool (Müller and Wehner, 1988). The ants use an odometer (Wittlinger et al., 2006) and optic flow (Pfeffer and Wittlinger, 2016) to determine the distance covered. By integrating the odometer information with information about the walked directions, for which the ants use the celestial compass (Müller and Wehner, 1988; Wehner et al., 1996; Wehner, 2003), they determine a vector pointing homewards. The celestial compass mainly relies on information about the position of the sun and the skylight polarization pattern in the UV-spectrum (Duelli and Wehner, 1973). This suggests that the skylight polarization pattern only in the UV-spectrum could provide a suitable reference system for the compass information of the path integrator to align gaze directions during learning walks.

The polarization direction of the UV-skylight is detected by specialized ommatidia in the dorsal rim area of the compound eye (Labhart and Meyer, 1999). The information is transferred by neurons forming the anterior optical tract (AOT) via several stages into the central complex (CX; Schmitt et al., 2016). In the CX polarization of the skylight is represented in a map-like pattern (Heinze and Homberg, 2007; Homberg et al., 2011; Heinze and Reppert, 2012). The CX was also shown to be involved in several tasks closely linked to orientation and navigation (Pfeiffer and Homberg, 2014; Fiore et al., 2017). In *Drosophila* the CX is additionally involved in landmark memory (Neuser et al., 2008), landmark orientation and angular path integration (Seelig and Jayaraman, 2015). Another prominent neuronal pathway in bees and ants, the anterior superior optical tract (asot), transfers visual information into the visual subregions of the mushroom bodies (MB; Gronenberg, 2001; Yilmaz et al., 2016). The MBs are centers for sensory integration, learning and memory. They undergo substantial neuronal changes when exposed first time to light (*Drosophila*: Barth and Heisenberg, 1997; *Apis*: Scholl et al., 2014; *Cataglyphis*: Seid and Wehner, 2009; Stieb et al., 2010, 2012) and during the formation of long-term memory (*Acromyrmex*: Falibene et al., 2015; *Apis*: Hourcade et al., 2010).

The duration of learning walk behaviors lasts for up to 3 days (Wehner et al., 2004; Stieb et al., 2012; Fleischmann et al., 2016). This correlates with the time needed for stable long-term memory formation (Menzel, 2001; Hourcade et al., 2010; Falibene et al., 2015; Scholl et al., 2015) and the time needed to induce neuronal changes in the visual subregions of the MBs after exposure to light pulses in *Cataglyphis fortis* (Stieb et al., 2010, 2012). Therefore, learning walks are perfectly suited to study brain-behavior-environment interactions. In this study, we restricted the input into the sky-compass of *Cataglyphis noda* during their early learning walks to ask, which reference system the ants use during this early learning phase to align their gaze directions. Ants that participated in the behavioral field experiments were subsequently used for neuroanatomical analyses. This allowed us to look at the interaction between the learning-walk behavior, the received information during these walks, as well as changes in the neuronal architecture in the terminal stages of two visual pathways, the CX and the MBs. The results suggest that natural skylight polarization information with the UV part of the light spectrum present induce structural changes in the CX and the MBs indicating their role in the initial calibration of visual pathways processing celestial information. However, exclusion of sky-compass information did not prevent *C. noda* from looking back towards their nest entrance suggesting, that celestial cues do not serve as the initial reference system for compass information during learning walks.

## Materials and Methods

### Animals

**Figure 1:**
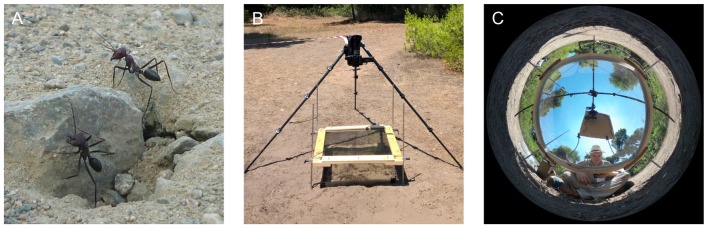
Experimental setup for skylight manipulation experiments. **(A)** Unmarked *C. noda* ants at the nest entrance. **(B)** 30 cm above the nest entrance, a filter was placed in order to alter the skylight information. Learning walks were recorded with a high-speed 4K-camera. In addition, a HD-camcorder recorded the nest entrance for the whole day. **(C)** Panoramic image of the UV-block with sunshade setup (UVBS). The observer was located in the south to trigger the high-speed recording and to prevent unmarked ants from leaving the area covered by the filter through the opening in the fence, which was located in the south-west.

Experiments were conducted with *C. noda* (Brullé 1832) (Figure 1A) in Schinias National Park, Marathonas, Greece from June–August 2016. A colony with a nest entrance in the middle of a small clearing in the pine forest of the national park (38°08’N 24°01’E) was used for the experiments. In order to make sure that only novices (ants performing learning walks for the first time) were used, all ants leaving the nest were marked on at least three consecutive days before the experiment using car paint (Motip Lackstift Acryl, MOTIP DUPLI GmbH, Haßmersheim, Germany). Unmarked ants can then be considered to be naïve, as it was shown in previous studies (Fleischmann et al., 2016, 2017). The animals were allowed to perform learning walks for three consecutive days within an arena (60 cm × 60 cm) restricted by a transparent plastic fence. Only marked foragers were allowed to leave the restricted area through a small exit in the fence.

### Manipulation of the Skylight

To manipulate the skylight the ants perceived during their learning walks, different filter systems (60 cm × 60 cm; Table 1) were placed 30 cm above the nest entrance from the third day of marking. Thereby, ants that did not leave the nest, but stayed inside of the nest entrance area would only perceive the altered skylight. The ants could still encounter the landmark panorama in the setup. As a control for the setup, a UV-permeable plexiglass was installed above the arena that did not alter the skylight perceived by the ants (UV100). To alter the skylight polarization pattern to an artificial, fixated one, a linear polarization filter was used. To test whether the full light spectrum without a polarization pattern had an influence on the ants’ behavioral development, a UV-permeable plexiglass that diffused the skylight was installed. The skylight polarization pattern and the position of the sun was blocked using a UV-impermeable plexiglass with a sunshade (UVBS; Figures 1B,C). On the second day of marking a camera set-up was placed north to the nest entrance. Two cameras were installed: a 4K-camcorder (HC-X1000, Panasonic Corporation, Kadoma, Japan) that recorded learning walks of novices at 50 fps, and a Full-HD camcorder (HDR-CX330E, Sony Corporation, Minato, Japan) that recorded the nest area at 25 fps for the entire day. Every time an unmarked ant left the nest entrance, an observer positioned south of the experimental setup triggered recordings of the 4K-camcoder using the Panasonic Image App (Version 10.9.2, Panasonic Corporation, Kadoma, Japan) on a Sony Xperia Z1 smartphone (Sony Corporation, Minato, Japan). Since it was not possible to film through the diffusor (Dif), only observational data is available for this experimental trial.

**Table 1:** Groups and filter systems used for skylight manipulation.

Group	Icon	Conditions	Analyses
DD	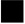	Interior workers that had not yet performed learning walks (excavated in the dark using red light);	Neuroanatomy
UV-Block with sunshade (UVBS)	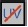	Three days of learning walks under a UV-light impermeable filter (Plexiglas (Gallery) 0A570 GT, Evonik Performance Materials GmbH, Essen, Germany) blocking 99.7% of the light below 420 nm with a sunshade, to additionally disguise the position of the sun;	Neuroanatomy Gaze direction
Diffusor (Dif)	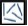	Three days of learning walks under a diffusor that lets UV-light pass (Plexiglas (GS) 2458 SC, Evonik Performance Materials GmbH, Essen, Germany), but diffuses any polarization pattern in the skylight;	Neuroanatomy
Polarization filter (P)	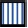	Three days of learning walks under a polarization filter (OUV6060-C—HNP’B replacement, Knight Optical Ltd., Harrietsham, United Kingdom) that lets UV-light pass, but provides an artificial linear, fixed polarization pattern;	Neuroanatomy Gaze direction
UV100	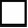	Three days of learning walks under a UV-light permeable Plexiglas (Plexiglas (GS) 2458, Evonik Performance Materials GmbH, Essen, Germany), as a control for the setup;	Neuroanatomy Gaze direction
No filter	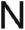	Three days of learning walks under natural conditions, as a control for the experiment;	Gaze direction

### Neuroanatomical Procedures

#### Anti-Synapsin Immunolabeling

On the third day of recording, novices that performed wide range learning walks reaching up the fence were captured under the filter setup and kept in the dark until the next day. This ensured, that the ants had performed several learning walks under the altered skylight conditions and that their brains had enough time to undergo structural changes (Stieb et al., 2012; Fleischmann et al., 2016, 2017; Schmitt et al., 2016). In addition, interior workers (DD) were collected from another nest in which, similar to the experimental nest, all ants leaving the nest were marked over three consecutive days. In order to get interior workers that had never seen daylight before, the nest was excavated in the night using red light. All ants were kept in a dark box until the next day.

To analyze neuroanatomical changes in the CX and MBs (all neuroanatomical nomenclature after Ito et al., 2014), the brains were stained using a primary antibody to synapsin (SYNORF1, kindly provided by E. Buchner, University of Würzburg, Germany) and a secondary antibody coupled to AlexaFluor 568 (A12380, Molecular Probes, Eugene, OR, USA) dye.

The ants were cooled down in a freezer and decapitated in the dark. Immediately afterwards the brains were carefully dissected and fixated in 4% formaldehyde in phosphate-buffered saline (PBS) for 1 day. The brains were then rinsed three times in PBS for 10 min, followed by one rinse in 2% Triton-X 100 solution in PBS and two rinses in 0.5% Triton-X solution, for 10 min each, to permeabilize cell membranes for antibody application on whole mount brains. To block unspecific binding sites, the brains were then incubated for 1 h at room temperature on a shaker in a 0.5% Triton-X 100 solution in PBS with 2% of Normal Goat Serum (NGS, Jackson ImmunoResearch Laboratories). Afterwards, the brains were incubated for 3 days in the refrigerator (~4°) on a shaker with the primary anti-synapsin antibody from mouse. A solution with 2% antibody, 2% NGS and 0.5% Triton-X 100 in PBS was used. After incubation the brains were rinsed five times for 10 min each in PBS. Then the secondary antibody, an anti-mouse antibody from goat with an Alexa Fluor 568 dye (4% in PBS with 1% NGS), was incubated for 2 days in the refrigerator on a shaker. The brains were then rinsed again three times in PBS for 10 min each, before they were dehydrated using an ethanol serial dilution. For that, they were rinsed for 10 min in every step: 30%, 50%, 70%, 90%, 95% ethanol in water and two times in 100% ethanol. The dehydrated brains were then cleared in methyl salicylate (M-2047; Sigma-Aldrich, Steinheim, Germany).

#### Anterograde Tracings of Neuronal Projections from the Medulla

To determine the neuronal projections via the asot in *C. noda*, projection neurons of the dorsal and ventral medulla (ME) were fluorescently stained in ants reared in laboratory colonies. The tracings of neuronal projections from the ME were performed using similar methods as described in detail in Yilmaz et al. (2016). Ants were cooled and fixed with clay. A small window was cut in the head capsule, and the brain was rinsed with cooled ant ringer solution. Using a thin glass capillary, dextran tetramethylrhodamine (micro-Ruby, D-7162, Molecular Probes, Eugene, OR, USA) and Dextran AlexaFluor488 (D-22910, Molecular Probes, Eugene, OR, USA) were focally inserted in the dorsal and ventral medulla. The brain was then rinsed with ringer solution and the head capsule was covered with a thin piece of Parafilm to prevent the brain from drying out. The dyes were allowed to be transported by incubating the ants for 3 h at room temperature in a dark box with high humidity. Afterwards, the brains were dissected in cooled ringer solution and fixated in 4% formaldehyde in PBS overnight. The brains were rinsed five times in PBS for 10 min each before they were dehydrated using an ethanol serial dilution. For that, they were rinsed for 10 min, each step: 30%, 50%, 70%, 90%, 95% in water, and two times 100% ethanol. The dehydrated brains were then cleared and mounted in methyl salicylate. Finally, the brains were digitized in the confocal laser scanning microscope (see below) using a 20×- or 10×-objective and step sizes of 5 μm or 10 μm.

### Data Analyses

#### High-Speed Video Analyses

The 4K-videos obtained from the experiments were converted into image stacks using the Free Video to JPG Converter (v. 5.0.99 build 823, DVDVideoSoft, DIGITAL WAVE LTD., London, UK). Subsequently, the pirouettes (tight back turns Fleischmann et al., 2017) performed by novices were analyzed frame by frame using the MATLAB (2015a, The MathWorks Inc., Natick, MA, USA) application DIGILITE (Jan Hemmi and Robert Parker, The Australian National University, Canberra, Australia). For this, the positions of the mandibles and the thorax were marked manually in each frame. Additionally, the position of the nest entrance and the north direction were marked. With these coordinates the gaze direction relative to the nest entrance of the ants during their back-turns was determined. The direction of the nest entrance was defined as 180°. Stopping phases during the pirouettes were defined as in Fleischmann et al. (2017), and the longest of these stopping phases was used to test the directedness of the back turns.

#### Neuroanatomical Analyses

For microscopic analyses, the brains that had been dissected and histochemically treated in our field laboratory were transferred to the University of Würzburg using a refrigerator unit (~4°C). A confocal laser scanning microscope (Leica TCS SP2, Leica Microsystems GmbH, Wetzlar, Germany) was used for scanning the brains as image stacks at a step size of 5 μm. We used the 10×-objective for overviews with 2.5 optical zoom NA imm (for CX), the 20×-objective with 2.7 optical zoom NA imm for the MB calyx, and the 63×-objective with 2.0 optical zoom NA imm for detailed scans in the lip (Li) and collar (Co) of the MB calyx. Subsequently, the volumes of the different components of the CX (fan-shaped body (FB), ellipsoid body (EB), protocerebral bridge (PB), noduli (No)) and of the MB calyx (Li, Co) were analyzed using the 3D-reconstruction software Amira (Amira 6.0.0, FEI Company, Hilsboro, OR, USA). In addition, synaptic complexes (microglomeruli, MG) were quantified in the visual and olfactory subregions of the MB calyx (Li, Co) using a modified version of the protocol by Groh et al. (2012; for further details, see Rössler et al., 2017). The CX, MB and other major neuropils were easily distinguishable in anti-synapsin labeled whole mount brains (Figure 3C), and based on tracings (Yilmaz et al., 2016, Figures 3A,B for *C. noda*). MB-calyx MG were quantified by counting the anti-synapsin labeled synaptic boutons in a defined volume of 1000 μm^3^. The MG density was then calculated by averaging multiple volumes of interest in the two subregions (three in the Co, four in the Li) as numbers of MG per μm^3^ following the protocol by Groh et al. (2012) and Muenz et al. (2015). From these numbers the total number of MG per calyx subdivisions was estimated by multiplying the MG densities by the volume of the corresponding neuropil. The ants used in this experiment had a median thorax length of 4.24 mm, ranging from 3.18 mm to 5.58 mm. Thorax length correlates with body size (Vowles, 1954) and, therefore, also with total brain size (Wehner et al., 2007). Since we did not find a correlation between thorax length and the analyzed neuropils of interest (Spearman roh test (*α* = 0.05): CX: *n*_CX_ = 45, *p*_CX_ = 0.545, *r*_CX_ = 0.093; MB: *n*_MB_ = 43, *p*_MB_ = 0.058, *r*_MB_ = 0.291), absolute volumes and MG numbers were used in this study. These results are coherent with results obtained using head width as a measure for body size in *C. fortis* (Stieb et al., 2010). As no major group-specific differences in thorax lengths were apparent (Supplementary Figure S1), comparisons were made without corrections for group bias in overall brain size.

**Figure 2:**
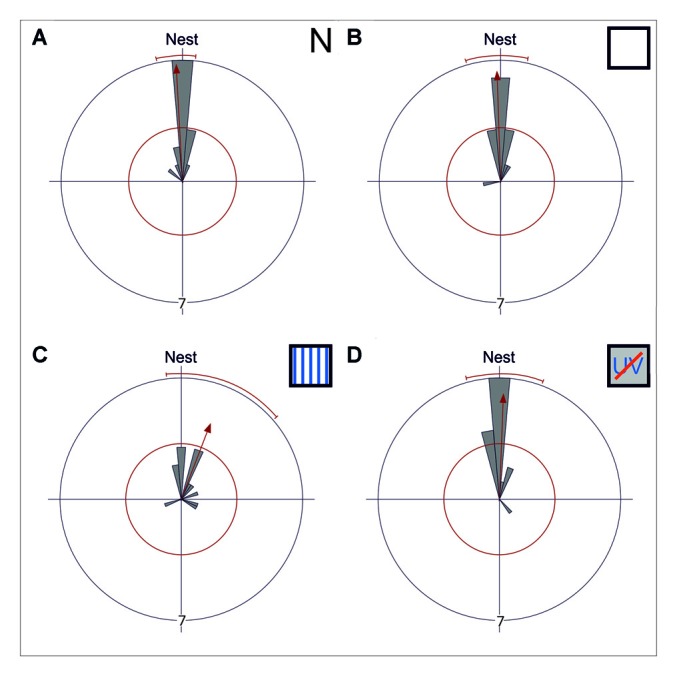
Gaze directions during the longest stopping phases under different skylight conditions. Data are shown in gray and the corresponding statistics in red. The bins of the circular histogram include 10 degrees. The red circle indicates the critical value *α* = 0.05 of the Rayleigh uniformity test. The red arrow indicates the r-vector pointing towards the mean direction. If the length of the vector exceeds the red circle the data is directed (*p* < 0.05). When the data is directed, a red line indicates the 95% confidence interval. If the expected direction (Nest ≙ 180°) lies within the confidence intervals limits, the data is directed towards the nest entrance. The outer circle indicates tic 7. Each data point is contributed by one back turn of one ant. **(A)** The mean gaze direction of the longest stopping phase in pirouettes during learning walks under natural/no filter conditions (N) is directed towards the nest entrance (*n* = 15). **(B)** The same is true for the mean gaze direction of the longest stopping phase under control conditions (UV100; *n* = 15) and **(C)** under an artificial, fixed polarization pattern (P; *n* = 14). **(D)** Even when excluded from all celestial information (UVBS; *n* = 15) the ants were able to gaze towards the nest entrance during the longest stopping phases. The mean angle and the angular variance did not differ between the four groups. For statistical details see text.

**Figure 3:**
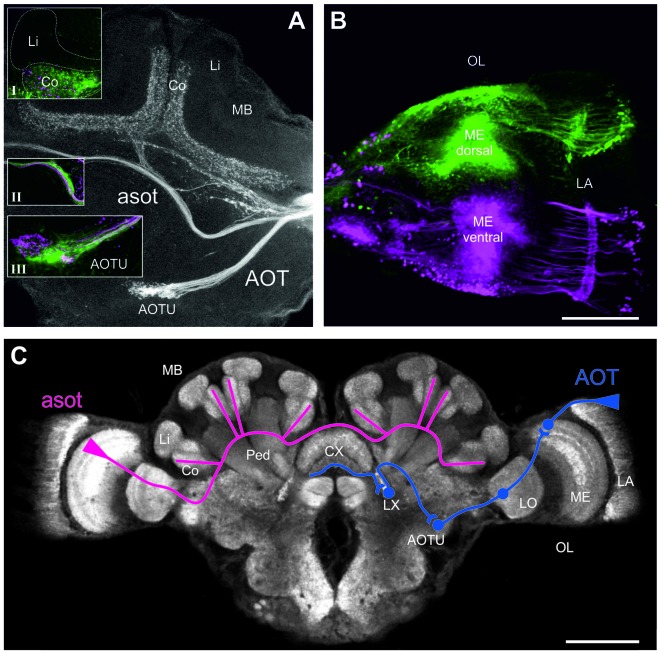
Neuronal projections from the medualla (ME) via the anterior optical tract (AOT) and the anterior superior optic tract (asot) in the *Cataglyphis noda* brain. Anterograde tracings from focal dye injections the dorsal and ventral medulla (ME; microruby in magenta, Alexa 488 dextran in green, see under **B**): **(A)** Axon bundles from projection neurons in the medulla run anterior above the peduncle (Ped) and the central complex (CX) into the visual subregion of the mushroom body (MB) collar (Co) on both sides of the brain. Axonal projections from both the dorsal and the ventral ME run along the asot (inset **II**) into the Co. The most prominent input in the MB-calyx Co was found in injections into the dorsal ME (green) compared to those in the ventral ME (magenta) (inset **I**). Axonal projection from the ME also run into the anterior optical tubercle (AOTU) along the AOT. Z-projection from a stack of 27 images, 10x objective, 5 μm step size. Insets were taken with a 20x objective, 5 μm step size. **(B)** In the dorsal ME Dextran AlexaFloun488 (green) was injected using a glass capillary. In the ventral ME Dextran Tetramethylrhodamine (micro-Ruby) (magenta) was injected using a glass capillary. Images taken with a 10x objective, step size of 10 μm, stack of 19 images, zoom 2.65. The scale bar in **(B)**, also valid for **(A)**, is 100 μm. **(C)** Schematic depiction of the tracing of the asot (magenta) and the AOT (blue). The asot, as seen in the tracings in **(A)**, runs from the ME anterior above the peduncle and the CX into Co. The AOT (information combined with the one from Schmitt et al., 2016) runs from the dorsal rim of the lamina (LA) to the dorsal rim of the ME, and from there via the LO to the AOTU to be relayed further to the lateral complex (LX). The anterior CX pathway terminates in the lower half of the ellipsoid body (EB) of the CX (Schmitt et al., 2016). The confocal scan of the *C. noda* brain shows an anti-synapsin labeled brain, similar to the staining procedure used for the neuroanatomical analyses. The scale bar is 200 μm.

#### Statistical Analyses

The gaze directions were grouped into 10°-bins as previously done by Fleischmann et al. (2017). The circular statistical software Oriana (Kovach Computing Services, Anglesey, UK) was used to check with the Rayleigh test whether the data was randomly distributed. If the gaze directions were directed (*α* = 0.05), we calculated the 95% confidence interval to check whether the expected direction (nest entrance: 180°) was within the limits. The mean angle and the angular variance were compared between the groups using a Mardia-Watson-Wheeler multisample test (*α* = 0.05).

In the neuroanatomical studies, the volume between the different groups (DD, UVBS, Dif, P, UV100) within each neuropil (CX, Co, Li) was compared using the Kruskal-Wallis-test (*α* = 0.05). In cases when a difference between the groups occurred, a *post hoc* pairwise comparison between DD and the other groups was performed using a Mann-Whitney U-test with Bonferroni correction. A critical value of *α* = 0.05 was used (after Bonferroni correction: *α* = 0.0125).

## Results

### Gaze Direction Analyses and Behavioral Observations Under Different Skylight Conditions

While initially leaving their nest under natural conditions (N), *C. noda* walked in small loops around their nest entrance, similar as shown earlier (Fleischmann et al., 2017). These learning walks were repeatedly interrupted by characteristic turns, so called voltes and pirouettes. During the latter, the ants performed multiple stopping phases (*n* = 15, 4 ± 1.75, median ± IQR) with the longest stopping phases directed towards the nest entrance (Rayleigh Uniformity Test: *Z*_0_ = 13.856, *n* = 15, *p* < 0.001; 95% Confidence Interval (−/+) 167.9°/186.0°; Mean: 177.0°; Figure 2A). The gaze direction during the longest stopping phases was directed towards the nest entrance when the experimental setup was installed using a UV-light permeable filter as a control (UV100; Rayleigh Uniformity Test: *Z*_0_ = 12.306, *n* = 15, *p* < 0.001; 95% Confidence Interval (−/+) 163.9°/192.55°; Mean: 178.2°; Figure 2B). When the natural skylight polarization pattern was altered to a linear one that did not change over the day (P) the overall structure of the walks remained unchanged and the gazes during the longest stopping phases were clearly directed towards the nest entrance (Rayleigh Uniformity Test: *Z*_0_ = 6.189, *n* = 14, *p* = 0.001; 95% Confidence Interval (−/+) 173.1°/229.1; Mean: 201.1°; Figure 2C). One analyzed pirouette under P did not contain a stopping phase and therefore was not included in the circular statistics. After the learning walks had taken place for several days under this fixed polarization pattern, the polarization filter was rotated by either by 90° or in two steps of 45°. From visual observations we noticed that the sudden changes in the polarization pattern above the nest entrance seemed to increase the number of naïve ants performing learning walks shortly after the change took place (experimental day with stationary linear polarization pattern number of learning walks: *n* = 71 vs. experimental day with stepwise rotated (45° every hour) linear polarization pattern number of learning walks: *n* = 277). When learning walks were performed under a diffused polarization pattern (Dif) no apparent changes in learning walk patterns compared to natural conditions could be observed. For the Dif conditions, further quantitative video analyses were not possible since we could not record through the diffusor. Nevertheless, more than 100 pirouettes, all directed towards the nest entrance, were observed during the three experimental days. However, even learning walks that were performed under the exclusion of any sky compass information by blocking UV-light, which is necessary for the ants to perceive the polarization pattern (Duelli and Wehner, 1973), and, at the same time, by excluding the position of the sun by using a sunshade (UVBS) were not altered in their overall structure compared to learning walks under natural conditions. The longest stopping phase of pirouettes under UVBS conditions was directed towards the nest entrance (Rayleigh Uniformity Test: *Z*_0_ = 11.406, *n* = 15, *p* < 0.001; 95% Confidence Interval (−/+) 166.4°/200.0°; Mean: 183.2°; Figure 2D). The mean angle or the angular variance did not differ between all experimental groups (Mardia-Watson-Wheeler multi sample test: *W* = 6.124; *n*_N_ = 15; *n*_UV100_ = 15; *n*_P_ = 14; *n*_UVBS_ = 15; *p* = 0.375).

### The AOT and asot in the *Cataglyphis* Brain

To investigate visual pathways to high-order integration centers in *C. noda* brains, we performed focal dye injections and anterograde neuronal tracings of neuronal projections from the dorsal and ventral medulla (ME; Figure 3B). This clearly revealed neuronal projections via the asot and via the AOT (Figure 3A). From 16 dye injected brains, three were successfully double stained (dorsal and ventral ME), three showed tracings from the dorsal ME only, and two from the ventral ME only. In all tracings, the asot projected from the ME anteriorly above the peduncle and the central complex (CX), bilaterally into the collar (Co) of the medial and lateral branches of the MBs (Figures 3A,C). Visual inspection of all tracings indicated that axonal projections via the asot from the dorsal ME were more prominent compared to the sparser projections and terminal branches from the ventral ME in the MB Co (*n* = 8; Figure 3A, inset I).

All tracings from the dorsal and ventral ME revealed projections to the anterior optic tubercle (AOTU) via the AOT (Figures 3A,C). The AOT was previously described in detail for *C. fortis* (Schmitt et al., 2016) by tracing projections only from the dorsal rim area of the lamina LA and ME. From there further stages are the lobula (LO), the AOTU, the lateral complex (LX) and finally the lower half of the EB of the CX (Figure 3C; for locust: Homberg et al., 2011; for *C. fortis*: Schmitt et al., 2016). Interestingly, our differential tracings from the dorsal and ventral ME revealed a clear pattern in the AOTU with a clear separation of ventral and dorsal projections in the upper unit of the AOTU and a mixed pattern in the lower part of the AOTU (Figure 3A, inset III).

### Influence of Manipulated Skylight Input during Learning Walks on Neuronal Plasticity in the CX and MB

**Figure 4:**
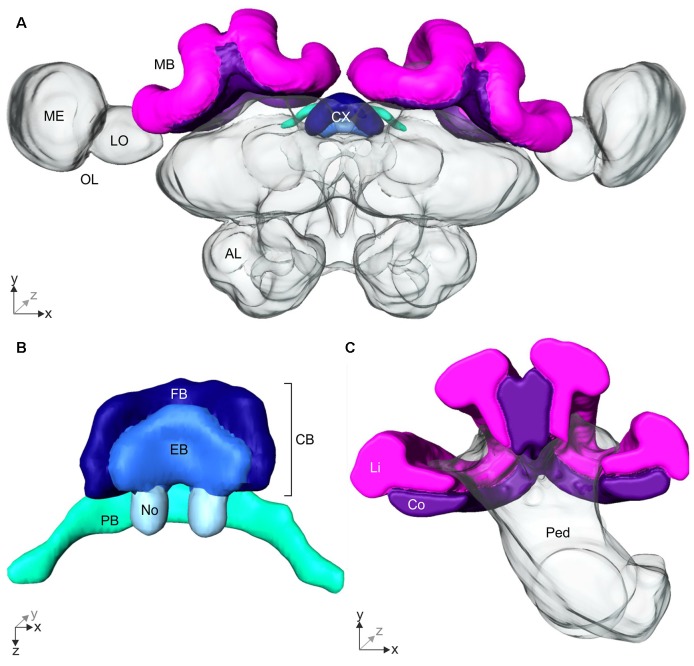
3D-reconstruction of the *Cataglyphis noda* brain. With the 3D-reconstruction software Amira the neuropils of the *C. noda* brain were manually reconstructed from the image stack obtained by the confocal laser scanning microscope. **(A)** 3D-reconstruction of a whole *C. noda* brain. To analyze the influence of celestial information during learning walks on neuroplasticity, the terminal stages of two visual pathways were reconstructed. The AOT transfers visual information, including polarization information, into the central complex (CX, shades of blue). The CX is located at the midline of the ant brain. Via the asot visual information is transferred to the mushroom body calyces (MB, magenta). Additionally, antennal lobes (AL), and optical lobes (OL) with the medulla (ME) and lobula (LO) are labeled. **(B)** The CX comprises several neuropils: The central body (CB, dark and light blue) is located most anterior. It consists of the large fan-shaped body (FB, dark blue) and the smaller ellipsoid body (EB, light blue), which is covered by the FB dorsally. Behind the CB, two globular neuropils, the noduli (No, pale blue) are located. Dorsally to that and slightly detached from the CB, the protocerebral bridge (PB, green) spans in a bridge-like shape between the mushroom bodies (MB). **(C)** The MB calyx includes the visual input region, the collar (Co, violet) and the olfactory input region, the lip (Li, magenta). They are located at the dorsal rim of the peduncle (Ped). Scale bars, **(A)** 200 μm; **(B,C)** 100 μm.

We investigated the influence of skylight manipulations during learning walks on neuronal changes in the terminal stages of the AOT and asot. The brains of ants that had participated in the behavior tests and had performed several days of learning walks under normal or altered skylight conditions were analyzed using 3D-reconstructions of the CX and MB (Figure 4), and quantifications of synaptic complexes in the MB. For comparison, brains of ants that had not yet performed learning walks (DD) were analyzed.

#### Volumetric Changes in the CX

**Figure 5:**
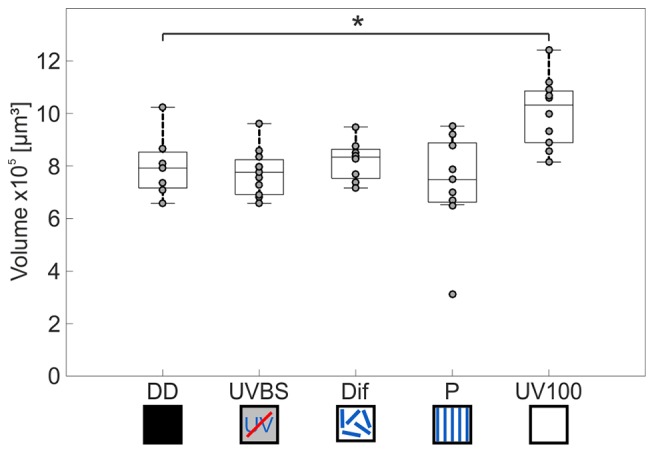
Volume changes of the CX after 3 days of learning walks dependent on celestial information. The central line of each boxplot depicts the median of the data. The upper and lower limits of the boxes show the 25th and 75th percentiles, while the whiskers extend to the extreme data points without outliers. All data points (including outliers) are plotted as gray circles. A difference between the groups can be found using a Kruskal–Wallis test. With the Mann-Whitney U-test with a Bonferroni correction the data was *post hoc* compared to the DD group. The asterisk indicates that data is significantly different (after correction *p* < 0.0125) from the DD group. The central complex shows a volume increase compared to interior workers (DD; *n* = 7) only if the ants perceived the UV mediated natural polarization pattern that changes over the day (UV100; *n* = 10). If the polarization pattern was altered, either by diffusion (Dif; *n* = 8) or by a linear polarization filter (P; *n* = 9), no change in the volume of the CX occurred compared to DD. Similarly, when ants were excluded from any celestial information (UVBS; *n* = 11), no volume increase occurred. For statistical details and further explanations, see text.

The AOT transfers visual information into the CX (Figure 3C). The CX comprises several neuropils (Figure 4B): The central body (CB) is located most anterior and consists of the large FB and the smaller EB, which is covered by the FB dorsally. Behind the CB, two globular neuropils, the No, are located. Dorsally to that and slightly detached from the CB, the PB spans in a bridge-like shape between the MBs (Figure 4B). Comparing the CX of the ants that had previously participated in the behavioral studies (DD, UVBS, Dif, P, UV100), showed a statistically significant difference between their CX volumes (Kruskal–Wallis test: CX Volume: $\chi_{4}^{2}$ = 16.38; *n* = 45; *p* = 0.0046; Figure 5). Compared to the CX of interior workers (DD) the CX in brains of *C. noda* that had performed several learning walks under a naturally changing polarization pattern (UV100) showed a volumetric increase (Mann-Whitney U-test with Bonferroni correction: DD vs. UV100, *Z*_4_ = −2.6837; *n*_DD_ = 7; *n*_UV100_ = 10; *p* = 0.0073). The volumetric increase in the CX was absent compared to DD when the ants performed their learning walks under restricted skylight conditions including an artificially fixed linear polarization pattern (P) (Mann-Whitney U-test with Bonferroni correction: DD vs. P, *Z*_3_ = 0.4234; *n*_DD_ = 7; *n*_P_ = 9; *p* = 0.6720), a diffused polarization pattern (Dif) (Mann-Whitney U-test with Bonferroni correction: DD vs. Dif, *Z*_2_ = −0.7522; *n*_DD_ = 7; *n*_Dif_ = 8; *p* = 0.4519), and without polarization pattern and information about the position of the sun (UVBS; Mann-Whitney U-test with Bonferroni correction: DD vs. UVBS, *Z*_1_ = 0.5434; *n*_DD_ = 7; *n*_UVBS_ = 11; *p* = 0.5869). The same statistical relationships were found for the volume of the CB only, which includes the ellipsoid (EB) and the fan-shaped body (FB). When comparing the subunits (EB, FB, PB and No) individually, the same tendency was found, but was not statistically significant.

#### Volumetric Changes in the MB and Plasticity of Synaptic Complexes

**Figure 6:**
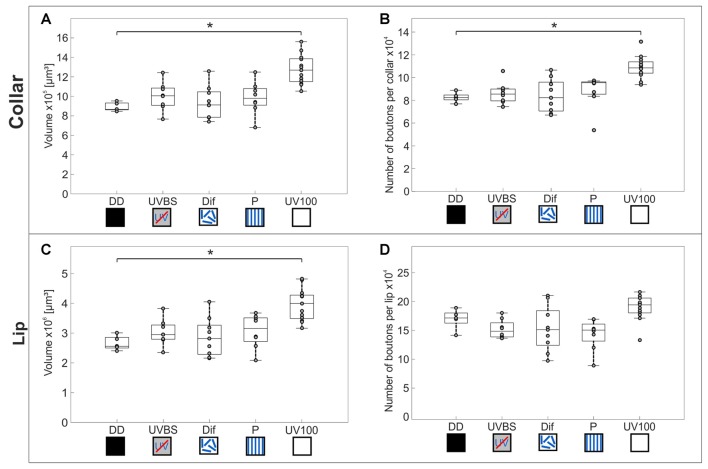
Volume changes and changes in numbers of synaptic complexes in MB calyx subdivisions. The central line of each boxplot depicts the median of the data. The upper and lower limits of the boxes show the 25th and 75th percentiles, while the whiskers extend to the extreme data points without outliners. All data points (including outliers) are plotted as gray circles. To find a difference between the groups, a Kruskal–Wallis test was used (*α* = 0.05). With the Mann-Whitney U-test with a Bonferroni correction the data was *post hoc* compared to the DD group if the Kruskal–Wallis test indicated a difference. The asterisk indicates data significantly different (after correction *p* < 0.0125) from the DD group. **(A)** In the MB-calyx Co, a significant volume increase compared to DD (*n* = 5) occurred only when the learning walks were conducted under the natural UV mediated changing polarization pattern (UV100; *n* = 13). **(B)** Similarly, the total number of synaptic boutons per calyx in the Co only increased when the learning walks were performed under UV100 compared to DD. **(C)** In the MB-calyx Li, a volume increase occurred only under UV100 conditions compared to DD, similar to the conditions in the Co. **(D)** However, in the Li no change in the total number of synaptic boutons per calyx occurred under any conditions. No significant differences in the MB occurred between DD and UVBS (*n* = 8), Dif (*n* = 9), or P (*n* = 8). For statistical details and further explanations, see text.

Comparison of the volume and the numbers of synapsin labeled synaptic boutons in the MB calyx Co (Figure 4C) revealed a significant difference between the experimental groups of the behavior essay (Kruskal–Wallis test: Co Volume: $\chi_{4}^{2}$ = 22.43; *n* = 43; *p* = 0.00016; Co No. Synapses: $\chi_{4}^{2}$ = 23.06; *n* = 43; *p* = 0.00012; Figures 6A,B). Only ants that had performed several learning walks under a naturally changing skylight polarization pattern (UV100) showed an increase in the volume of the MB calyx Co and the estimated total number of synapses per calyx compared to ants that had not yet performed learning walks (DD; Mann-Whitney U-test with Bonferroni correction: Co Volume: DD vs. UV100, *Z*_4_ = −3.1543; *n*_DD_ = 5; *n*_UV100_ = 13; *p* = 0.0016; Co No. Synapses: DD vs. UV100, *Z*_4_ = −3.1543; *n*_DD_ = 5; *n*_UV100_ = 13; *p* = 0.0016). All groups that had performed learning walks under restricted skylight conditions did not show a significant increase compared to DD, neither in the volume nor in the total number of MG synaptic complexes per calyx in the MB calyx Co (Mann-Whitney U-test with Bonferroni correction: CO Volume: DD vs. UVBS, *Z*_1_ = −1.5370; *n*_DD_ = 5; *n*_UVBS_ = 8; *p* = 0.1243; DD vs. Dif, *Z*_2_ = −0.2667; *n*_DD_ = 5; *n*_Dif_ = 9; *p* = 0.7897; DD vs. P, *Z*_3_ = −1.5370; *n*_DD_ = 5; *n*_P_ = 8; *p* = 0.1243; Co No. Synapses: DD vs. UVBS, *Z*_1_ = −0.6587; *n*_DD_ = 5; *n*_UVBS_ = 8; *p* = 0.5101; DD vs. Dif, *Z*_2_ = 0; *n*_DD_ = 5; *n*_Dif_ = 9; *p* = 1; DD vs. P, *Z*_3_ = −1.8298; *n*_DD_ = 5; *n*_P_ = 8; *p* = 0.0673). The volume of the Li also differed significantly between groups (Kruskal–Wallis test: Li volume: $\chi_{4}^{2}$ = 20.08; *n* = 43; *p* = 0.00048; Figure 6C). The volume was increased significantly compared to DD in ants that had performed several learning walks under UV100 conditions (Mann-Whitney U-test with Bonferroni correction: Li Volume: DD vs. UV100, *Z*_4_ = −3.1543; *n*_DD_ = 5; *n*_UV100_ = 13; *p* = 0.0016). No difference in the Li volume occurred between DD and the other groups, (Mann-Whitney U-test with Bonferroni correction: Co Volume: DD vs. UVBS, *Z*_1_ = −1.2443; *n*_DD_ = 5; *n*_UVBS_ = 8; *p* = 0.2134; DD vs. Dif, *Z*_2_ = −0.5333; *n*_DD_ = 5; *n*_Dif_ = 9; *p* = 0.7897; DD vs. P, *Z*_3_ = −1.5370; *n*_DD_ = 5; *n*_P_ = 8; *p* = 0.1243). In contrast to the visual MB subregion (Co), however, there was no significant difference compared to DD based on pair-wise comparison of the total number of MG synaptic complexes per calyx in the MB olfactory Li, despite the groups not coming from the same distribution (Kruskal–Wallis test: Li No. synaptic complexes: $\chi_{4}^{2}$ = 15.81; *n* = 43; *p* = 0.0033; Mann-Whitney U-test with Bonferroni correction: Li No. synaptic complexes: DD vs. UVBS, *Z*_1_ = 1.5370; *n*_DD_ = 5; *n*_UVBS_ = 8; *p* = 0.1243; DD vs. Dif, *Z*_2_ = 0.9333; *n*_DD_ = 5; *n*_Dif_ = 9; *p* = 0.3506; DD vs. P, *Z*_3_ = 1.9762; *n*_DD_ = 5; *n*_P_ = 8; *p* = 0.0481; DD vs. UV100, *Z*_4_ = −1.9715; *n*_DD_ = 5; *n*_UV100_ = 13; *p* = 0.0487; Figure 6D).

## Discussion

### Celestial Information Is Not Necessary for the Look Back to the Nest Behavior

In the beginning of their foraging careers*, C. noda* perform learning walks that are repeatedly interrupted by turns with several stopping phases. The longest stopping phases are accurately directed towards the nest entrance (Fleischmann et al., 2017). It has previously been suggested that ants may use path integration information to align their back turns (Graham et al., 2010; Müller and Wehner, 2010). We used this conspicuous feature in the learning walks of *C. noda* as an easily quantifiable behavior readout in skylight manipulation experiments to ask whether celestial cues may serve as a reference system to align gaze direction. Our results demonstrate that neither an artificial (P) nor a diffused (Dif) polarization pattern disturbed the directedness of the longest stopping phases toward the nest entrance. Even with complete exclusion of the polarization pattern and the position of the sun (UVBS), the ants were still able to perform the look back to the nest entrance behavior. This strongly suggests that the celestial compass—providing the directional component of the path integration system during foraging (review: Wehner, 2003)—is not the system of reference used by ants to initially align the gaze direction during naïve learning walks. Our results underline the robustness and importance of the mechanism that is used to align the gaze direction during the longest stopping phases.

### Possible Reference Systems for the Look Back to the Nest Behavior

As our results show that the celestial compass does not provide a reference system used during learning walks, other possibilities for the compass component of the path integrator have to be considered. A potential candidate could be the visual landmark panorama. Schultheiss et al. (2016) recently demonstrated that UV-light plays a crucial role for the use of the landmark panorama. However, our results show that *C. noda* was still able to look back to the nest entrance during learning walks under blocked UV-light spectrum (UVBS). Furthermore, the panorama information is not yet known or memorized in ants during naïve (first) learning walks and requires the completion of several learning walks (Fleischmann et al., 2016). The ants might also use nest odors to detect the direction of the nest. *C. fortis* were shown to use olfactory landmark cues near the nest (Steck et al., 2009). However, as the ants conduct their learning walks in increasing distances and in all compass (including upwind) directions away from the nest (Fleischmann et al., 2016), olfactory cues are not reliable during the entire learning walk sequences. The ants also walk cross wind in order to approach odor sources, in particular prey items during foraging (Wolf and Wehner, 2000; Buehlmann et al., 2014). This behavior has not become evident in learning walks, and as the ants perform pirouette-like turns all-around the nest entrance, cross wind orientation seems highly unlikely. Finally, the ants could use intrinsic (idiothetic) orientation mechanisms. Such mechanisms however, would be highly prone to cumulative errors (Müller and Wehner, 1994). An error in the gaze direction during the longest stopping phase of pirouettes would lead to a snapshot taken into the wrong direction. This could easily lead to serious errors in foragers, but also during learning walks with extended lengths. A more promising candidate for an initial reference system for the compass component of the path integrator during learning walks of *C. noda* is the geomagnetic field. This had already been suggested for the learning flights of bumblebees (Collett et al., 2013). Furthermore, *C. noda* was shown to learn magnetic landmarks (Buehlmann et al., 2012). Although the magnetic field strength, in these experiments, was far above the natural geomagnetic field, it appears likely that the ants possess a magnetic sense that could be used for the initial calibration of navigational information. A potential role of a magnetic sense has also been suggested for other ants (fire ants: Anderson and Vander Meer, 1993; leaf-cutter ants: Banks and Srygley, 2003; wood ants: Çamlitepe and Stradling, 1995; for a review see: Wajnberg et al., 2010). However, so far no use of the geomagnetic field for navigation, in particular as compass information for path integration, has been described in ants, neither for experienced foragers during their foraging runs, nor for learning walks in novices. Therefore, at this point the question regarding an initial reference system for the alignment of gaze directions to acquire and calibrate navigational information during learning walks has to remain open.

### Visual Pathways in the *C. noda* Brain

To be used as navigational information, the visual information perceived by the ants during learning walks needs to be relayed to and processed in higher integration centers of the brain. Using anterograde tracing techniques, two prominent visual pathways become apparent in *C. noda*. Visual information from the ME is transferred bilaterally to the MB collars of the medial and lateral MB calyces, very similar to the projections found in other Hymenoptera (Gronenberg, 2001; Yilmaz et al., 2016). In *Drosophila* only a very small subset of visual neurons transfers information from the OL to the MB calyx (Vogt et al., 2016). This may suggest that this pathway is highly conserved across insects, but the number of neurons and their projection patterns are adapted to the visual ecology of individual species (Groh et al., 2014; Vogt et al., 2016; Yilmaz et al., 2016). One interesting feature in *C. noda* is that axonal projections from the dorsal ME appear more extensive compared to projections from dye injections into the ventral ME. This may indicate that the dorsal retina and celestial view aspects are more prominently represented in the MB calyx Co compared to terrestrial aspects from the lower part of the compound eye. More focal injections, also along the horizontal axis, are needed to further analyze this. In *C. fortis* the AOT was shown to house projections from the dorsal most regions of the medulla indicating that polarization information from the dorsal rim area of the eye is transferred via this pathway to the AOTU and the LX into the lower half of the EB of the CX (Schmitt et al., 2016), similar to the conditions found in locusts (Homberg et al., 2011). Our results show that also the ventral region of the medulla is relayed to the upper and lower part of the AOTU. Next we tested whether the high-order sensory integration centers (MB, CX) express neuroplasticity related to the quality of celestial information experienced during learning walks.

### Natural Polarization Pattern Is Necessary for a Volume Increase in the CX

Although our manipulations of celestial information did not significantly alter the learning walk behavior, the restriction of skylight information interfered with neuroanatomical changes in the CX. A volume increase in the CX as compared to DD occurred only when the learning walks had been conducted under the full spectrum including UV-light and the naturally changing polarization pattern. Exposure to the full light spectrum including UV-light with an artificial, fixed polarization pattern (P) or without a usable polarization pattern (Dif) did not lead to a CX volume increase. In contrast, a volume increase in the CB of *Drosophila* occurs after the flies were exposed to UV-light (Barth and Heisenberg, 1997). However, in that case *Drosophila* did not perceive a natural light and polarization pattern. In *C. noda* the exclusion of UV-light, and thereby the reception of the polarization pattern during learning walks, prevented volumetric chances of the CX. It is not possible with the methods available to count synapses within subunits of the CX. Therefore, we only analyzed volumetric changes in the CX. Previous studies on large synaptic complexes (giant synapses, GS) in the lateral complex (LX) along the sky-compass pathway of *C. fortis* revealed a significant increase of GS numbers depending on exposure to the UV part of the light spectrum (Schmitt et al., 2016). Therefore, it seems likely that the volume increase in the CX is also due to an increase in the number of synapses along this pathway. This increase was found to be significant in the CB units, i.e., the input region of the CX. Within the CX, in particular the PB, the skylight polarization direction is represented in a map-like manner (Pfeiffer and Homberg, 2014), and it has been shown through computational investigation that the CX is able to store spatial information (Fiore et al., 2017). Whether the neuroanatomical changes we found in the CX are triggered by appropriate sensory exposure or following the formation of spatial memory is an interesting question that needs to be investigated in a more focused approach. The CX is also involved in higher order control of movement of the limbs (Strauss, 2002; Martin et al., 2015), landmark orientation, and angular path integration (Seelig and Jayaraman, 2015). All this makes the CX a well suited neuropil to link polarization information to other stimuli mediating directional information important for navigation, for example other terrestrial reference systems.

### Sensory Experience of a Natural Polarization Pattern Is Necessary for an Increase in the Number of Synaptic Complexes in the Visual Subregions of the MB Calyx

Similar to the results just described for the CX it was only under exposure to the naturally changing UV polarization pattern that a volume increase was found in the MB-calyx of ants that had performed their learning walks. Kühn-Bühlmann and Wehner (2006) had previously shown an increase in the MB volume of experienced (aged) foragers compared to dark reared ants of age-controlled *Cataglyphis bicolor*. In our study, we focused on the transition phase between interior worker (DD) and forager. Our data suggests that a volume increase in the MBs occurs already during learning walks and that it is dependent on the presence of the natural polarization pattern (UV100). A net increase of MB synaptic complexes was found only in the visual input region. As the MB is a higher order integration center involved in learning and memory, this may indicate that the increase in MG numbers is related to visual experience. Computer simulations by Ardin et al. (2015) suggest that the large synaptic capacity of visual subregions in ant MBs are well suited for the storage of visual snapshots underling the potential role of the MBs for learning and memorizing panoramic landmark cues during learning walks. Studies by Stieb et al. (2010, 2012) have shown that the MB Co expresses light-induced and age-dependent changes in MG numbers in *C. fortis*. Stieb et al. (2010) also showed a volume increase in the Co after exposure to full spectrum light accompanied by a decrease in MG densities. Furthermore, studies in the honeybee (Hourcade et al., 2010) and leafcutter ants (Falibene et al., 2015) showed that the formation of stable long-term olfactory memory leads to an increase in the density and number of MG in the Li. In contrast to the laboratory and partly restrained conditions in these experiments, the ants used in our study were allowed to perform their natural behaviors in their natural habitat under natural or altered skylight conditions. Therefore, a mix of both effects—the first exposure to light and long-term memory formation following learning, might be expected in our experimental ants. As UV-light is crucial for learning terrestrial landmarks (Schultheiss et al., 2016), an increase in synaptic complexes could be expected in the presence of UV-light, even without a naturally changing polarization pattern (Dif). Our data shows that a volume increase in the Co was absent in ants that had performed their learning walks under the full light spectrum, but without a usable polarization pattern (Dif) or with an artificial, fixed polarization pattern (P). Only when ants perceived a full spectrum including UV light together with a naturally changing polarization pattern, an increase in the volume and number of MG occurred in the MB calyx Co. No such effect was seen in MB collar MG of honeybees after a fine color discrimination task (Sommerlandt et al., 2016) indicating that only certain parameter combinations may lead to measurable effects of structural synaptic plasticity. The increase in the estimated MG numbers in the MB-calyx Co indicates an outgrowth of new presynapses during learning walks under natural skylight—a process similar to what has been observed after the formation of long-term memory (Hourcade et al., 2010; Falibene et al., 2015).

Due to the prominent role of path integration, *Cataglyphis* have to calibrate their internal skylight compass to the solar ephemeris (the season- and place-specific course of the sun over the day) at the beginning of their foraging career (Wehner and Müller, 1993). A panoramic- and celestial snapshot based mechanisms based on long-term memory in the visual MBs might play a role in this initial calibration. Similarly, short term learning of celestial snapshots was recently suggested for sky-compass orientation in dung beetles (el Jundi et al., 2016). When the skylight polarization pattern, however, does not change over the day (P), is diffused (Dif), or is not available (UVBS), it would not make sense to take and store celestial snapshots. To store new celestial information and thereby fine tune an internal template of the solar ephemeris function makes only sense if the polarization pattern changes compared to a fixed reference system. This hypothesis is also backed up by our observation that the number of learning walks drastically increased when the linear polarization filter was rotated. Analyzing neuroanatomical changes in ants that have performed learning walks under such a systematically changed artificial polarization pattern would allow for a deeper insight into the correlation shown so far.

The present study represents a first step of probing potential effects of learning walks on neuroplasticity. We started this combined field and laboratory study by focusing on the terminal projection areas of two prominent visual pathways in the CX and MB. To obtain a more comprehensive understanding of the total extent of learning-walk induced neuroplasticity, future investigations will have to include more extensive neuroanatomical analyses of all major brain neuropils, for example analyzing their volume relationships, synapse densities (whenever feasible), also in relation to overall brain volumes—for example like it was done in recent volumetric analyses of brains in migratory and solitary locusts, or migratory and non-migratory moths (Ott and Rogers, 2010; de Vries et al., 2017). In the same line, as previous work in *Camponotus* ants (Yilmaz et al., 2016) and in *Drosophila* (Barth and Heisenberg, 1997) show that the optic lobes undergo plastic changes after artificial light exposure, future studies on learning-walk induced neuroplasticity in *Cataglyphis* ants should include neuropils peripheral to the CX and MB, like the optic lobes, the AOTU and the lateral complex.

## Conclusion

Neither the polarization pattern, or other information from UV-light input, nor the position of the sun are necessary for *C. noda* to align their gaze directions during the longest stopping phase of pirouettes in learning walks. Thus, the celestial compass as part of the path integrator does not provide the ants with the reference system needed during naïve learning walks. However, although not being necessary for the accuracy of the look-back behavior, we show that proper perception of the natural polarization pattern that changes over the day is important for triggering neuroanatomical changes in the CX and MB calyx that take place during learning walks. In the MB-calyx Co, this volume increase is linked to an increase in the number of MG synaptic complexes indicating that plasticity related processes are triggered when the ants are confronted with a naturally perceived polarization pattern that changes over the day.

## Ethics Statement

This study was carried out in accordance with the Greek and German laws.

## Author Contributions

RG, PNF, RW, WR conceived the study. WR and RW led the study. RG, PNF and KG collected and analyzed the data. RG and PNF drafted and WR and RW revised the manuscript. All authors approved the final version of the manuscript for submission.

## Conflict of Interest Statement

The authors declare that the research was conducted in the absence of any commercial or financial relationships that could be construed as a potential conflict of interest.
