# Supplementary material for: The Role of Celestial Compass Information in Cataglyphis Ants during Learning Walks and for Neuroplasticity in the Central Complex and Mushroom Bodies
Source: Front Behav Neurosci. 2017 Nov 14;11:226. doi: 10.3389/fnbeh.2017.00226 (PMC5694495; doi:10.3389/fnbeh.2017.00226)
Supplement: Supplementary file 1 [file Data_Sheet_1.docx]

Supplementary Material

The role of celestial compass information in *Cataglyphis* ants during learning walks and for neuroplasticity in the central complex and mushroom bodies

Robin Grob^*^, Pauline N. Fleischmann^*^, Kornelia Grübel, Rüdiger Wehner, Wolfgang Rössler

*** Correspondence:**Robin Grob: robin.grob@uni-wuerzburg.de
Pauline N. Fleischmann: pauline.fleischmann@uni-wuerzburg.de

*These authors contributed equally to this study.

# Supplementary Figure

**
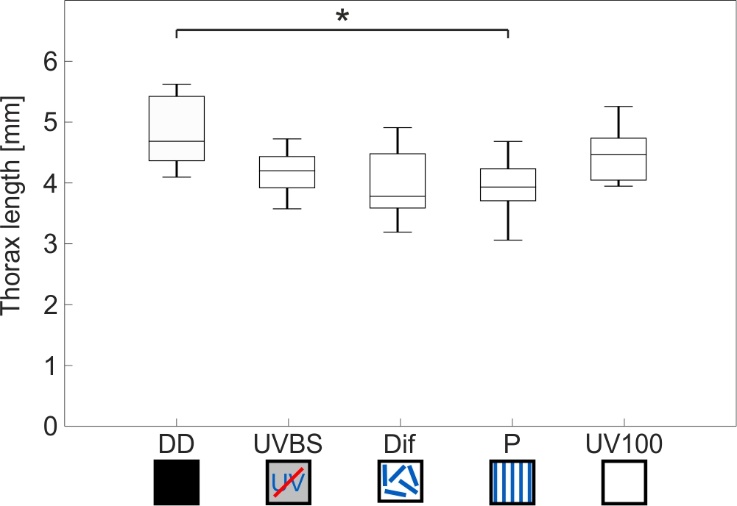
**

**Supplementary Figure 1.** **Comparison of the thorax length between the experimental groups.** The central line of each boxplot depicts the median of the data. The upper and lower limits of the boxes show the 25th and 75th percentiles, while the whiskers extend to the extreme data points without outliers. A difference between the groups can be found using a Kruskal–Wallis test (χ_4_^2^=11.62; n=49; p=0.0240). The data was post-hoc compared to the DD group using the Mann-Whitney U-test with a Bonferroni correction. The asterisk indicates that data is significantly different (after correction p<0.0125) from the DD group. Only between DD and P the thorax length significantly differed (Mann-Whitney U-test with a Bonferroni correction: DD vs. P, Z_3_=2.5404; n_DD_=7; n_P_=9; p=0.0118). No difference was found between the other groups compared to DD (DD vs. UVBS, Z_1_=2.2642; n_DD_=7; n_UVBS_=11; p=0.0260; DD vs. Dif, Z_2_= 2.1170; n_DD_=7; n_Dif_=9; p= 0.0418; DD vs. UV100, Z_4_=2.5404; n_DD_=7; n_UV100_=13; p= 0.1779). Since no correlation between thorax length and the volume of the neuropils of interest was found, no correction for the allometric differences was used for the further analyses.
